# How IT preparedness helped to create a digital field hospital to care for COVID-19 patients in S. Korea

**DOI:** 10.1038/s41746-020-00366-4

**Published:** 2020-12-03

**Authors:** Se Young Jung, Ho-Young Lee, Hee Hwang, Keehyuck Lee, Rong-min Baek

**Affiliations:** 1 Office of eHealth Research and Business, Seoul National University Bundang Hospital, Seongnam-si, Republic of Korea; 2 Department of Family Medicine, Seoul National University Bundang Hospital, Seongnam-si, Republic of Korea; 3 Department of Nuclear Medicine, Seoul National University Bundang Hospital, Seongnam-si, Republic of Korea; 4 Department of Pediatrics, Seoul National University Bundang Hospital, Seongnam-si, Republic of Korea; 5 Department of Plastic Surgery, Seoul National University Bundang Hospital, Seongnam-si, Republic of Korea

**Keywords:** Palliative care, Health policy

## Coronavirus disease 2019 (COVID-19) outbreak in S. Korea

**Fig. 1:**
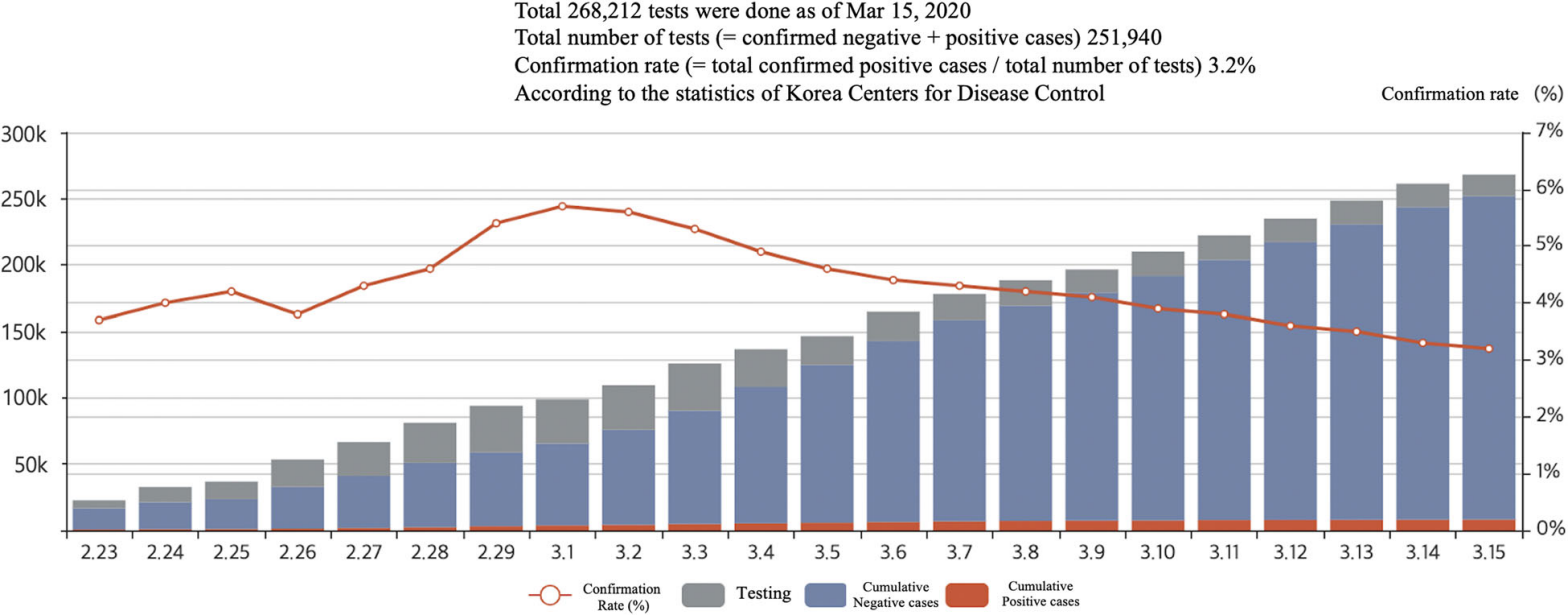
Status of COVID-19 diagnostic tests in S. Korea. According to Korea Centers for Disease Control, total 268,212 COVID-19 tests were done as of 15 March 2020. Data and figure derived from the Korea Centers for Disease Control.

South Korea has overseen one of the worst COVID-19 outbreaks outside China with over 8162 confirmed cases reported by 15 March 2020. The government succeeded in curbing the spread of the disease until mid-February. However, the number of confirmed cases exponentially increased since mid-February, when the number of cases increased because of a secretive religious organization in Daegu, changing the strategy from containment to mitigation of COVID-19, as the government failed to prevent mass infection via intensive quarantine and extensive epidemiological intervention (Fig. 1).

In Daegu, the medical system was at the brink of collapse. Although the number of new patients with COVID-19 increased rapidly, patients waiting to be admitted suddenly deteriorated and died at home. In order to prevent the collapse of the entire medical system, the government started to operate “community treatment centers (CTCs).” In addition, the government applied the standards for hospitalization in the national negative pressure care units more strictly.

CTCs are facilities that are under the care of the daily medical staff. Before the admittance of patients with COVID-19, the principle was that all individuals infected with COVID-19 would be hospitalized and treated in a negative pressure room, regardless of their risk. However, after experiencing mass dissemination of COVID-19 in Daegu, the Korean government revised the triage strategy to prevent the depletion of medical resources.

## CTC as a “digital field hospital”

**Fig. 2:**
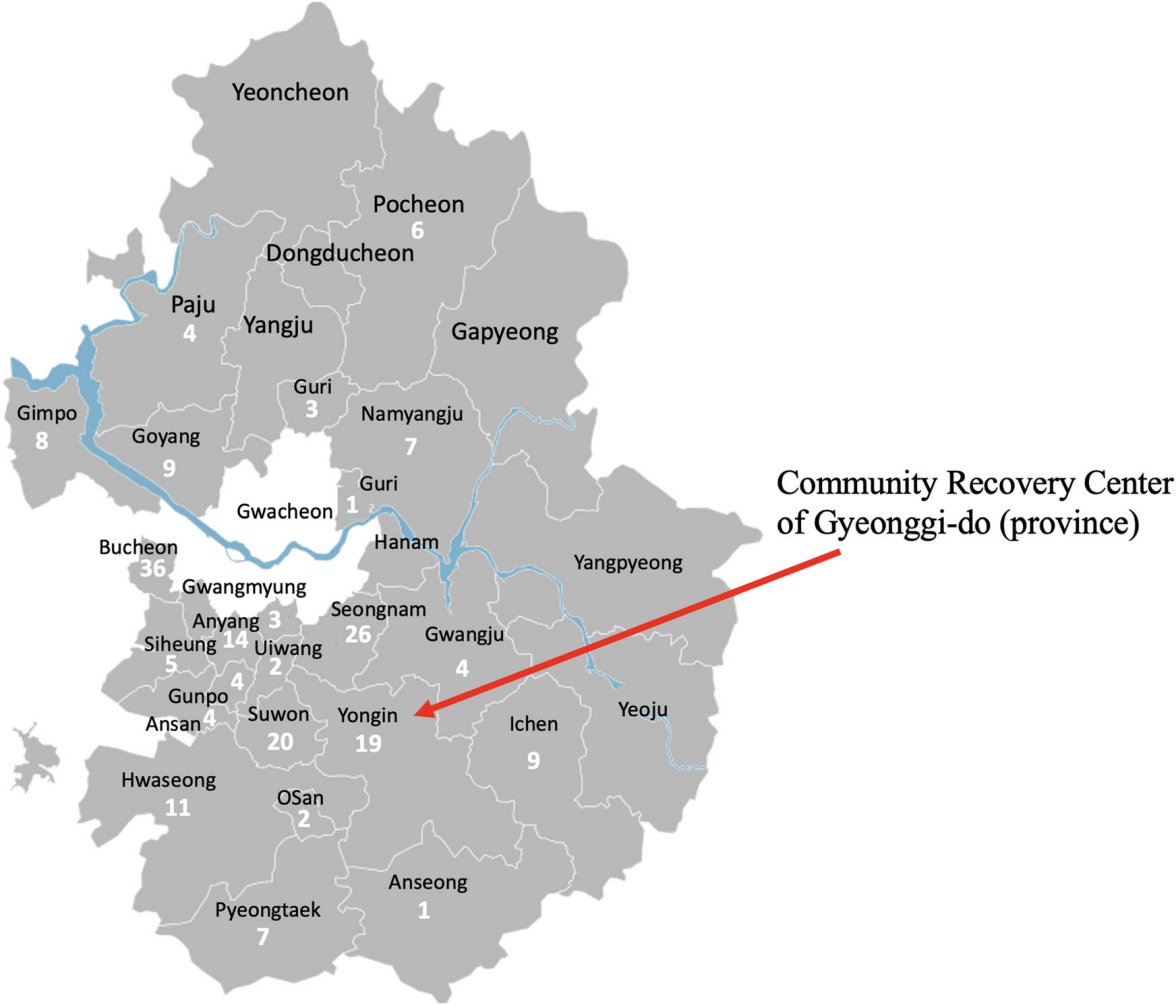
Status of confirmed cases in the 32 districts of Gyeoggi-do. Gyeonggi-do had 205 confirmed cases of COVID-19 in the 32 districts as of 15 March 2020. Data and figure derived from the Korea Centers for Disease Control. Figure modified from Wikimedia Commons (https://commons.wikimedia.org/wiki/File:Gyeonggi_Municipal.svg), under the Creative Commons Attribution-Share Alike 3.0 Unported License.

Gyeonggi-do, the second-largest local government after Seoul, started operating CTCs in Yongin to prevent medical resource depletion, as observed in Daegu, in advance (Fig. 2). Patients who had almost recovered, but still tested positive for reverse transcription-polymerase chain reaction, and low-risk patients waiting for hospital admission with mild symptoms were enrolled in CTCs.

In the CTCs, the faculty of family medicine, dispatched from Seoul National University Bundang Hospital (SNUBH), was present 24 h to take care of patients. Patients admitted to the CTCs reported vital signs and symptoms at regular intervals according to the national guidelines.

### IT preparedness of SNUBH helped create a digital field hospital to manage COVID-19

**Fig. 3:**
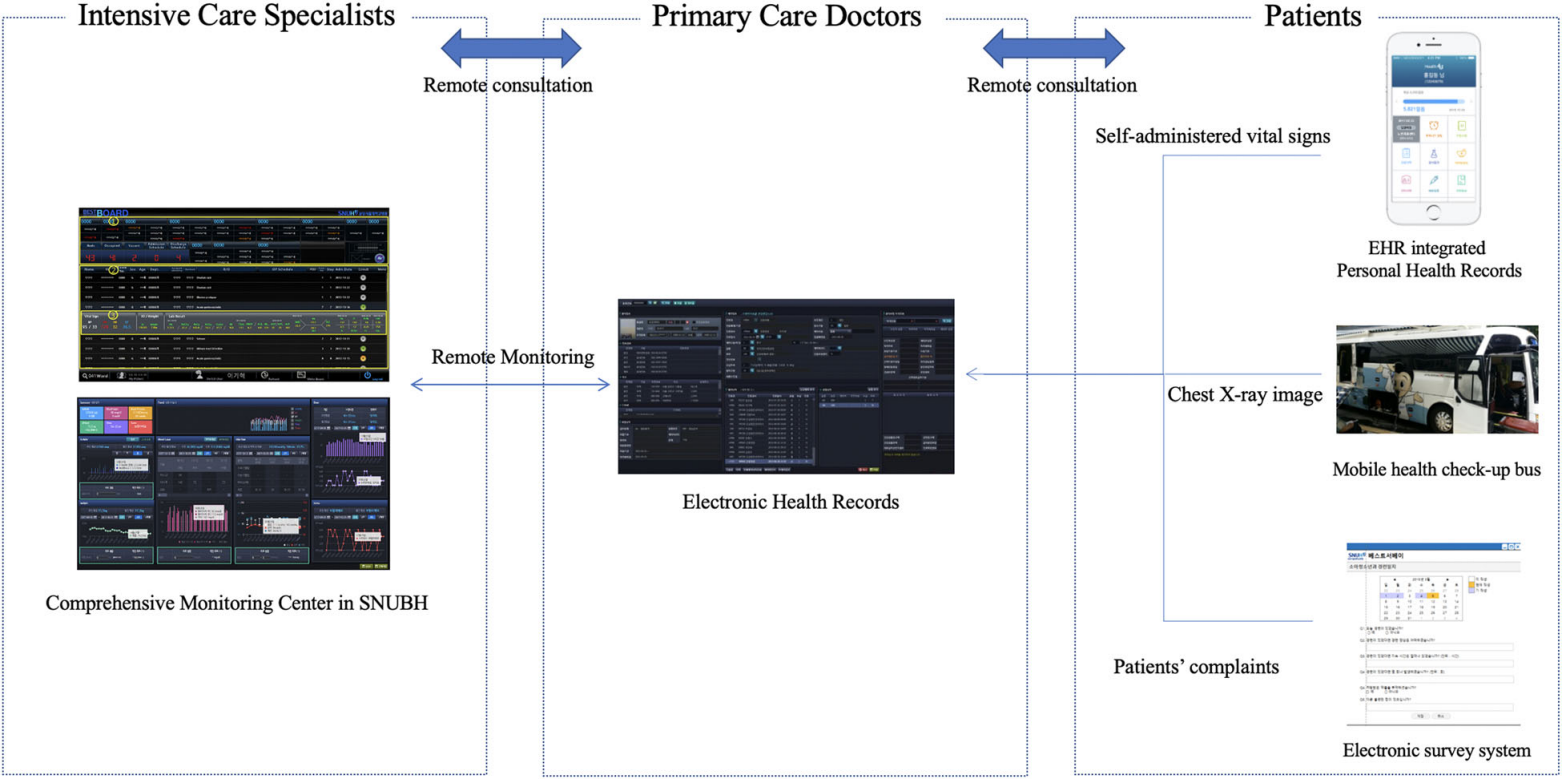
The overall architecture of telemedicine for community treatment center supported by SNUBH. Intensivists and primary care doctors can monitor patients in the community treatment center through telemedicine. Figure derived from Seoul National University Bundang Hospital.

When developing an unprecedented medical system, it is essential to design the system with the fewest resources, with as little damage to the current medical system as possible. SNUBH’s IT preparedness made it possible to create a fully functional “digital field hospital” in a short time without wasting medical resources. When designing a CTC as a digital hospital with only a few medical staff on-site, we took three important considerations into account. First, we minimized direct contact with the patient. Second, all information was recorded electronically and incorporated into electronic health records (EHRs). Finally, we implemented a telemonitoring system to intervene effectively when necessary. Based on SNUBH’s IT technology, we could quickly build a CTC digital hospital for the COVID-19 outbreak (Fig. 3).

### EHRs

SNUBH was established in 2003 as the first paperless hospital in the Asia Pacific area, which has over 500 beds. Since its inception, SNUBH has utilized the homemade EHR, BESTCare. In 2010, it became the first hospital outside the USA to receive the Healthcare Information and Management Systems Society stage 7 certification and succeeded in recertification in 2016 and 2019. BESTCare integrates all the patients’ data bidirectionally through an electronic monitoring system based on a 55-inch touchscreen dashboard and personal health records. In the CTCs, BESTCare was utilized as an information hub to gather all the medical information from the patient. Patients in the CRC had video calls with the medical staff every morning, like the doctors’ roundup in the hospital. The medical staff entered and managed the information obtained during the video call directly into the EHR.

### Telemonitoring via the electronic dashboard

SNUBH has been operating the EHR-integrated dashboard, BESTBoard, that was implemented as a rapid response system (RRS) in 2012^1,2^. Through the device, healthcare professionals can access all the information on EHRs optimized 55-inch touchscreen. The system can be utilized for monitoring the vital signs of all the patients in a ward on one screen. During monitoring, the same screen can be used to query all EHR information of a patient for quick decision making. We implemented the system as a remote electronic intensive care unit in a conference room of SNUBH. Intensive care specialists took turns conducting telemonitoring. An early warning system helped RRS users make the right decisions promptly.

### Integration of vital signs via personal health records (PHRs) tethered to EHRs

**Fig. 4:**
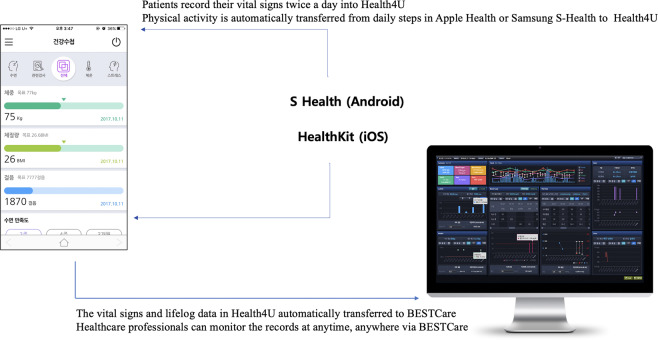
The overall process of integrating vital signs from Health4U to BESTCare. Patients in the community treatment center can integrate vital signs through Health4U to BESTCare. Figure derived from Seoul National University Bundang Hospital.

It was essential to establish a process to measure the vital signs without contacting the patient. We used SNUBH’s PHR solution. SNUBH launched a homegrown PHR application named Health4U in 2013^3–6^. Health4U communicates with BESTCare, using Health Level 7 Fast Healthcare Interoperability Resources. Furthermore, Health4U has been integrating patient-generated health data (PGHD), including vital signs such as blood pressure, pulse rate and body temperature. PGHD can also be entered manually by the patient, and it can also automatically receive records from Apple Health or Samsung S-health via Bluetooth^7^ (Fig. 4). Vital signs may not be entered automatically when the Bluetooth communication interferes with other radio waves, or the Bluetooth function is accidentally turned off, so we adopted a method in which patients enter their body temperature, pulse, and blood pressure in Health4U manually. The PHR was developed mainly to manage patients with chronic diseases. However, we fully utilized it to monitor the vital signs of COVID-19 patients in CTCs.

### Integration of patients’ complaints via electronic surveys

SNUBH has been conducting surveys since 2015 through an electronic survey system. The result of the survey is automatically integrated into BESTCare. The system was used to collect the main symptoms in the CTCs. The symptoms recorded by the patient were automatically integrated into the EHR so that the medical staff could periodically check the patient’s condition. Also, a clinical decision support system in the electronic survey alerted a risk when a “risk sign” was spotted in the information entered by the patient.

## Implications

The healthcare IT technology introduced and used by SNUBH since 2003 has been fully utilized to provide value-based care that reduces medical costs and improves patient health outcome. In particular, SNUBH continuously improved the quality of medical treatment and research by analyzing extensive data collected from all the IT infrastructure and transferred to the EHR^8^. The IT systems such as PHR and electronic survey system were mostly designed to manage noncommunicable chronic diseases. However, paradoxically, it is most effectively used to manage highly contagious communicable diseases such as the COVID-19 outbreak, which represents the possibility that healthcare ICT can be flexibly changed for various medical situations.

Unlike the United States that has already embraced the telemedicine revolution, S. Korea is one of the countries that have made telemedicine illegal^9^. Korea is one of the world’s leading countries in the industry; however, the Korean government has failed to address the long-standing demand in the healthcare industry that would ease telemedicine regulations^10^. Nevertheless, the telemedicine technology required in emergencies, such as COVID-19 outbreak, has been maturing in many ways, and the technology introduced by SNUBH for the CTC operation showed us how IT readiness of hospitals and healthcare systems would help patients and healthcare professionals in such an unexpected nationwide disaster.

The COVID-19 pandemic revealed the real value of telemedicine. However, telemedicine is not achieved in 1 day. As observed in the case of SNUBH, the IT preparedness is crucial when facing a disastrous new infectious disease, such as COVID-19. Depending on the nation, there are considerable gaps in healthcare IT. Nonetheless, medical institutions and governments around the world need to invest in healthcare IT infrastructure to prepare for unpredictable situations that may prevail in the future.
